# In Vitro Anticancer Effects of Aqueous Leaf Extract from *Nepeta nuda* L. ssp. *nuda*

**DOI:** 10.3390/life14121539

**Published:** 2024-11-24

**Authors:** Zlatina Gospodinova, Georgi Antov, Svetozar Stoichev, Miroslava Zhiponova

**Affiliations:** 1 Institute of Plant Physiology and Genetics, Bulgarian Academy of Sciences, “Acad. Georgi Bonchev” Str., Bl. 21, 1113 Sofia, Bulgaria; antov8107@abv.bg; 2 Institute of Biophysics and Biomedical Engineering, Bulgarian Academy of Sciences, “Acad. Georgi Bonchev” Str., Bl. 21, 1113 Sofia, Bulgaria; svetlio85@abv.bg; 3 Department of Plant Physiology, Faculty of Biology, Sofia University “St. Kliment Ohridski”, 8 Dragan Tsankov Blvd., 1164 Sofia, Bulgaria

**Keywords:** *Nepeta nuda* L., anticancer potential, MDA-MB-231, MCF7, HT29, Colon 26, HepG2, anti-clonogenic effect, apoptosis, molecular targets

## Abstract

Despite significant efforts, cancer remains the second leading cause of mortality worldwide. The medicinal plant *Nepeta nuda* L. represents a valuable source of biologically active compounds with pharmacological activities including antioxidant, anti-inflammatory, antimicrobial, and antiviral. This study aimed to assess the antiproliferative potential and mechanisms of action of aqueous extract from the leaves of wild-grown *N. nuda*. Cancer cell lines, MDA-MB-231, MCF7 (breast), HT29, Colon 26 (colon), and HepG2 (liver cancer), and a non-cancerous skin cell line, BJ, were assessed for antiproliferative activity by MTT assay and observation of cell morphological alterations. The cancer cell line that was most sensitive to the extract was further studied for apoptotic alterations by Annexin V/propidium iodide staining, colony-forming assay, and qRT-PCR analysis. The results revealed that the plant extract inhibited the proliferation of all investigated cancer cell lines with the strongest cytostatic effect on Colon 26 cells with a half maximal inhibitory concentration (IC_50_) value of 380.2 μg/mL and a selectivity index (SI) of 3.5. The extract significantly inhibited the ability of cells to form colonies, exhibited considerable proapoptotic potential involving the participation of the *CASP8* gene, and increased the expression levels of *ATG3* and the *BECN1* gene, which suggests a role of autophagic cell death in the antitumor action.

## 1. Introduction

Cancer is a leading cause of mortality globally, accountable for nearly one in six deaths [1]. According to the World Health Organization (WHO), in 2022, there were about 20 million new cases, and a considerable increase to about 35 million new cases is predicted by 2050. Breast, colon, and liver cancer are among the most commonly diagnosed cancer types in the world and take second (2,308,897 new cases or 11.6% of all cases worldwide), third (9.6%), and sixth (4.3%) places, respectively. Regarding malignant diseases-related deaths, the second position is occupied by colon cancer with 903,859 death cases (9.3% of all cancer deaths), followed by liver (7.8%) and breast cancer (6.9%).

Due to the adverse side effects of standard cancer therapy, the scientific community is increasingly turning its attention to plant-based cancer treatment options. Offering an extraordinary variety of biologically active substances with diverse functions, plant species are relatively safe sources of new specific and reliable medicinal substances.

*Nepeta nuda* L., also known as “hairless” catmint, is a herbaceous perennial plant that belongs to the genus *Nepeta* (fam. Lamiaceae). Primarily distributed in Central and Southern Europe, the Middle East, Central and Southern Asia, and parts of Africa, *N. nuda* is also naturalized in Northern Europe, New Zealand, and North America [2,3]. Considering the wide climatic range and altitude of distribution, the plant is a rich source of bioactive compounds, some of which are phenolic derivatives (protocatechuic, chlorogenic, rosmarinic, and ferulic acids; aesculin; apigenin and rutin), terpenoid derivatives (camphene, nerolidol, and camphor), etc. [4,5,6,7].

Thanks to its exceptional chemical composition, the medicinal herb is used not only in traditional but also in modern medicine, as lotion against snake and scorpion bites; a palliative against syphilis, diuretic, and bronchodilator; and a sedative, as well as for stomach disorders and rheumatic pains, etc. [8,9,10,11]. In addition to the applications already mentioned, numerous studies have reported the presence of anti-inflammatory, wound-healing, antiviral, antibacterial, virucidal, antiprotozoal, and other properties in various *N. nuda* subspecies [12,13,14,15].

The literature data on the antitumor properties of *N. nuda* extracts are extremely scarce [16,17], and at present, no information is available about the antineoplastic potential of *N. nuda* L. ssp. *nuda*. Antitumor properties were reported for water, ethanol, and methanol extracts from the aerial parts of *N. nuda* L. subsp. *annua* var. *annua* collected from Turkey, investigated through an *Agrobacterium tumefaciens*-induced potato disc tumor assay [16]. Another study detected the cytotoxic potential of *N. nuda* subsp. *lydiae* essential oil from Turkey against Caco-2 colon cancer cells with an LD_50_ (median lethal dose) value of the oil of 129.6 µg/mL [17].

For this reason, the first objective of the present work is to assess the antitumor potential of aqueous leaves extract of wild-grown *N. nuda* L. ssp. *nuda*. The extract action on five cancer cell lines originating from some of the most common and lethal cancer types: breast (MDA-MB-231, MCF7), colon (Colon 26, HT29), and liver (HepG2) cancer, is assessed to select the cell line with the highest sensitivity and selective index. Subsequently, the mechanisms of action of the *N. nuda* extract anticancer activity in the cancer cell line most sensitive to the extract, including cytostatic capability and proapoptotic qualities, and identification of target molecules are investigated.

## 2. Materials and Methods

### 2.1. Plant Material and Extraction Procedure

The plant *N. nuda* L. ssp. *nuda* was collected from natural habitats in the Pirin mountain, Bulgaria, during the flowering period. A voucher specimen (SO108017) was deposited in the Herbarium of Sofia University “St. Kliment Ohridski”, Sofia, Bulgaria.

As described previously [6], the air-dried plants were stored in the dark at room temperature. The *N. nuda* phytochemical content has been previously investigated by UHPLC-LTQ OrbiTrap XL, 2.7.2 and UHPLC/qqqMS2 (Table S1, Figure S1). For the extraction, only leaves were ground into a powder and subjected to subsequent maceration at 60 ± 5 °C for 24 h at a ratio of 1 g dry weight to 10 mL solvent (water). The aqueous extract was filtered, and the solvent was evaporated as the frozen filtrate was lyophilized at −65 °C (Alpha 1-2 LDplus, Martin Christ Gefriertrocknungsanlagen GmbH, Osterode am Harz, Germany).

### 2.2. Cell Line and Cultivation Conditions

In this work, human breast cancer cell lines, MCF7 (luminal A breast cancer subtype) and MDA-MB-231 (triple negative breast cancer subtype) (American Type Culture Collection—ATCC, Manassas, VA, USA); a human colorectal carcinoma cell line, HT29 (ATCC, USA); a murine colon adenocarcinoma cell line, Colon 26 (CLS, Germany); a human liver cancer cell line, HepG2 (ATCC, USA); and a human non-cancerous skin cell line, BJ (ATCC, USA) were used. MDA-MB-231, HT29, Colon 26, HepG2, and BJ cells were cultivated in Dulbecco’s Modified Eagle’s Medium (DMEM) supplemented with 10% fetal bovine serum (FBS) in a humidified atmosphere containing 5% CO_2_ at 37 °C. For the cell line MCF7, insulin (0.01 mg/mL) was added to DMEM cell culture complete medium, containing 10% FBS.

### 2.3. MTT Cell Proliferation Assay

The cellular metabolic activity and cell proliferation rate were evaluated by MTT [3-(4,5-dimethylthiazol-2-yl)-2,5-diphenyl tetrazolium bromide] assay [18]. Briefly, cells (MCF7, MDA-MB-231, HT29, Colon 26, HepG2, and BJ) were seeded in 96-well plates at a density of 5 × 10^3^ cells/well and, on the next day, were treated with increasing concentrations of the *N. nuda* aqueous extract (10–600 μg/mL) for 72 h. As a negative control, untreated cells cultured in a medium were used. During the last 4 h of the treatment period, 20 μL MTT solution at a concentration of 5 mg/mL was added, and the samples were incubated in the dark. The formazan complex was then dissolved in 10% SDS and 0.01 M HCI, and the absorbance was measured at 570 nm on a microplate reader (Thermo Scientific Multiskan Spectrum, Waltham, MA, USA).

The percentage of cell proliferation was calculated using the following formula:

(1) $$Cell\;proliferation\left(\%\right)=\left(Absorbance\;treated\;sample/Absorbance\;control\right)\times 100$$

The values of the half-maximal inhibitory concentrations (IC_50_) of the extract for the different cell lines were calculated using GraphPad Prism 8 (GraphPad Software, San Diego, CA, USA).

The degree of selectivity in the antiproliferative action of *N. nuda* extract was quantified by calculating the selectivity index (SI) according to the following formula:

(2) SI=IC50 non−cancerous cell line/IC50 cancer cells line

### 2.4. Light Microscopy of Cell Morphology Changes

The monitoring of alterations in the monolayer and morphology of MCF7, MDA-MB-231, HT29, Colon 26, HepG2, and BJ cells 72 h after treatment with *N. nuda* extract in a variety of concentrations was performed at magnification of 20× using an inverted microscope (Leica DMI3000 B, Leica Microsystems GmbH, Wetzlar, Germany and Carl Zeiss, Jena, Germany) equipped with a digital camera.

All the subsequent analyses were carried out on the most sensitive to the action of the extract cell line.

### 2.5. Colony-Formation Assay

The ability of single cells to reproduce into a colony of at least 50 cells was evaluated after seeding of cells at a density of 1 × 10^3^ cells per well in 6-well plates, incubated overnight, and then treated with *N. nuda* extract for 7 days. The experimental time of 7 days was chosen in accordance with the cell line characteristics and was optimal for the formation of visible well-differentiated colonies in the untreated controls. The colonies were visualized after fixation and staining with 2% methylene blue in 50% ethanol and then were counted under a microscope.

### 2.6. Fluorescence Microscopy Analysis of Apoptosis

To assess the potential of *N. nuda* extract to induce programmed cell death (apoptosis and necrosis) in cancer cells, a fluorescence microscopic analysis was performed by staining with Annexin V/propidium iodide (PI). Cells were placed at a density of 2 × 10^5^ per well in 6-well plates and, on the next day, were exposed to the action of the extract for 72 h. Afterward, cells were detached with a 0.25% trypsin-EDTA solution, collected, centrifugated for 5 min at 200× *g*, and washed twice with phosphate buffer saline (PBS). As a final step, incubation with Annexin V and PI was carried out in the dark for 15 min at room temperature, according to the instruction of the Annexin-V-FLUOS Staining Kit (Roche, Mannheim, Germany), and the samples were observed under a fluorescence microscope (Olympus BX-41, Olympus Europa GMBH, Hamburg, Germany).

### 2.7. Quantitative Reverse Transcription-Polymerase Chain Reaction (qRT-PCR) Analysis of Gene Expression

Analysis of the expression of specific genes, with a key role in the initiation of programmed cell death and in the control of the cell cycle and cell proliferation was performed by qRT-PCR analysis. For the analysis, cells treated for 72 h with *N. nuda* extract were harvested by trypsinization, centrifuged for 5 min at 200× *g*, washed with ice-cold PBS, and centrifuged again at 250× *g* for 5 min at 4 °C. RNA isolation was performed with a GeneJET RNA Purification Kit (Thermo Scientific Inc., Vilnius, Lithuania), according to the manufacturer’s protocol, with on-column DNase digestion using an RNase-Free DNase Set (Qiagen, Hilden, Germany). The purity and concentration of the extracted RNA were quantified with a BioSpec-nano Spectrophotometer (Shimadzu Biotech, Kyoto, Japan). Subsequently, 1 µg total RNA from the control and each sample was subjected to first-strand complementary DNA (cDNA) synthesis using a First Strand cDNA Synthesis Kit (Thermo Scientific Inc.) and random hexamer primers.

The quantitative RT-PCR analysis was performed using a 1x Luna^®^ Universal qPCR Master Mix (New England Biolabs Inc., Frankfurt am Main, Germany) on a PikoRealTM Real-Time PCR System (Thermo Fisher Scientific Inc., Waltham, MA, USA). For amplification, initial denaturation at 95 °C for 15 min was applied, followed by 45 cycles at 95 °C—15 s, 60 °C—30 s, 72 °C—45 s, and melting curve analysis (55–95 °C, with a temperature step of 0.2 °C). Data were analyzed using PikoReal Software version 2.2 (Thermo Fisher Scientific, Waltham, MA, USA). The Pfaffl equation [19] was used to quantify the relative expression fold changes of the genes of interest (*p53*, *BAX*, *CASP3*, *CASP8*, *BECN1*, *ATG3*, *MYC*, and *CDKN1A*) relative to the endogenous control (*β-actin*).

### 2.8. Statistical Analysis

All results are presented as mean ± standard error of the mean (SEM) from at least three independent experiments. Statistical significance between the untreated control and treated groups was determined by one-way ANOVA, followed by Dunnett’s post hoc test using GraphPad Prism 8, and the values at *p* < 0.05 were considered statistically significant. Correlation analysis was performed by SigmaPlot 11.0.

## 3. Results

### 3.1. Cell Proliferation Inhibitory Effect of N. nuda Extract

The anticancer properties of aqueous leaf extract from *N. nuda* enriched in valuable compounds ([6], Table S1, Figure S1) were studied on a panel of five cancer cell lines—breast (MDA-MB-231, MCF7), colon (Colon 26, HT29) and hepatocellular carcinoma (HepG2). The cancer cell lines were compared to the non-cancerous skin cell line BJ by MTT cell proliferation assay after 72 h treatment period in a wide range of concentrations.

**Figure 1:**
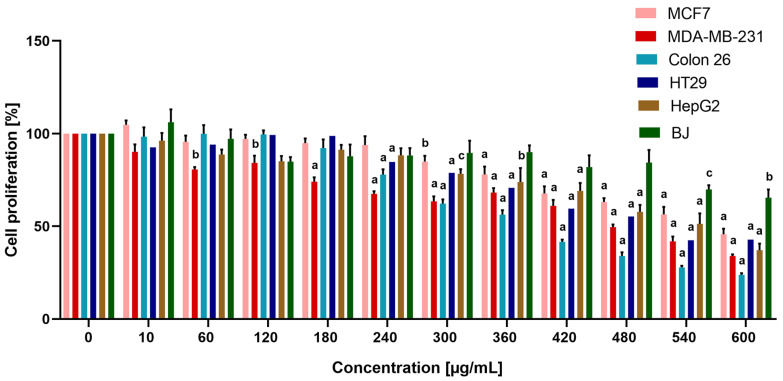
MTT cell proliferation assay of MCF7, MDA-MB-231, Colon 26, HT29, HepG2, and BJ cells treated with increasing concentrations of *N. nuda* aqueous leaf extract for 72 h. Error bars represent standard error of the mean (SEM). a, b, and c indicate significant differences from the control group by one-way analysis of variance (ANOVA) followed by Dunnett’s post-hoc test (a *p* < 0.0001, b *p* < 0.001, c *p* < 0.01).

The obtained results showed that the extract inhibited statistically significant growth of all of the investigated cancer cell lines with the strongest cytostatic effect on Colon 26 cells (Figure 1) with a maximal reduction of proliferation to 23.88% at 600 μg/mL. The weakest cytostatic effect of the studied extract was registered for the MCF7 breast cancer cell line. The extract exerted a slighter reduction in cell proliferation of the non-cancerous cell line BJ compared to cancer cells, and the estimated antiproliferative selectivity showed the highest selectivity index (SI), 3.5, regarding the Colon 26 cell line. The obtained values for the IC_50_ concentrations and selectivity index are described in Table 1. A statistical correlation was performed between the previously reported bioactive constituents (polyphenols and flavonoids), the antiradical activity of the aqueous extract [6], and the IC_50_ values reported in Table 1. The analysis showed strong interdependence between total polyphenols and the inhibition of Colon 26 tumor cell proliferation (Table S2).

### 3.2. Cell Morphological Alterations Caused by N. nuda Extract

**Figure 2:**
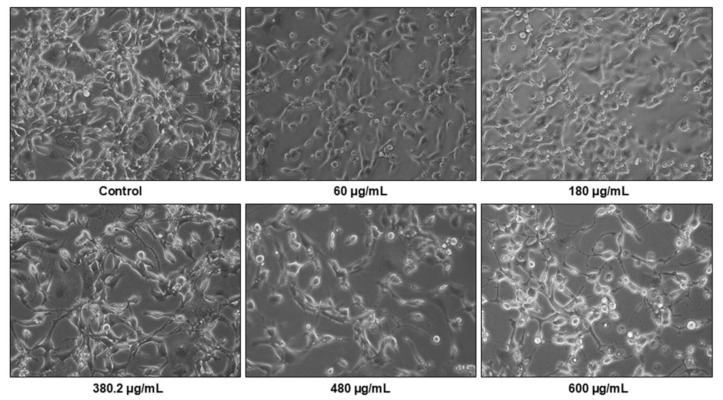
Morphological changes of Colon 26 cells after treatment for 72 h with 60, 180, 380.2, 480, and 600 μg/mL of *N. nuda* aqueous leaf extract compared to the untreated control. Magnification 20×.

To visualize possible changes in cell morphology and monolayer after exposure to the action of medicinal plant *N. nuda*, an observation under an inverted light microscope was carried out, which indicated alterations in the tumor cells, including rounding and reduction of the volume of the cells (Figure 2). Considerable decrease in the density of the cell monolayer after extract treatment were also observed in cancer cell lines.

**Figure 3:**
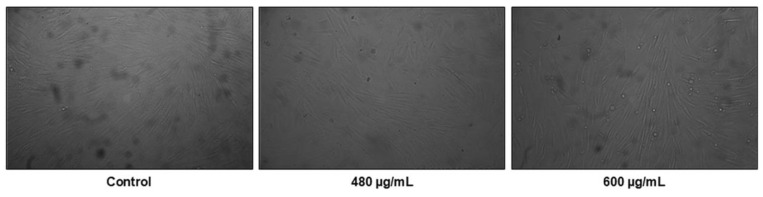
Morphological changes of BJ cells after treatment for 72 h with 480 and 600 μg/mL of *N. nuda* aqueous leaf extract compared to the untreated control. Magnification 10×.

Some distinguishable changes in cell morphology and monolayer density of non-cancerous cells BJ were found only after treatment with the highest tested concentration of plant extract of 600 μg/mL (Figure 3).

Based on the obtained results as a model system for further studies of the mechanisms of action of the *N. nuda* extract, colon cancer line, Colon 26, was chosen, showing the highest sensitivity towards the medicinal plant.

### 3.3. Clonogenic Potential of N. nuda Extract on Colon Cancer Cells

**Figure 4:**
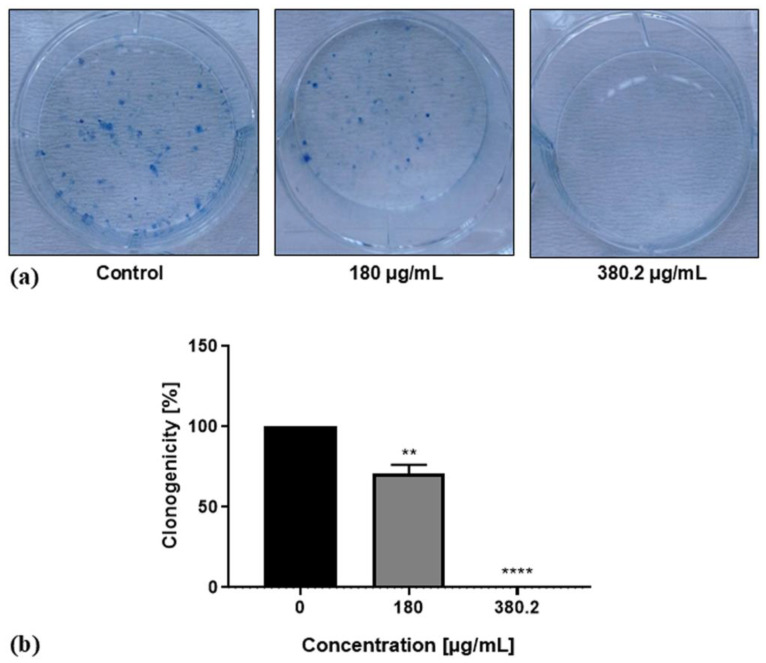
Effect of *N. nuda* aqueous leaf extract on clonogenicity of Colon 26 cells. (**a**) Effect of treatment for 7 days with 180 and 380.2 μg/mL extract on cancer cells when compared to the untreated control; (**b**) Quantitative evaluation of Colon 26 cells clonogenicity. Error bars represent standard error of the mean (SEM). ** *p* < 0.01, **** *p* < 0.0001 vs. the control group.

Colony-formation assay for the evaluation of the proliferative capacity of a single cell was accomplished after treatment of Colon 26 cells with the leaf extract of the plant for a period of 7 days (Figure 4).

A strong concentration-dependent anti-clonogenic effect of the plant substance was detected, while in the untreated control cells, well-formed colonies were observed. Applying the extract at a concentration of 180 µg/mL led to a reduction in the tumor cell clonogenicity to 70.63%, while the treatment of the cells with the detected IC_50_ concentration of the extract of 380.2 μg/mL resulted in complete inhibition of cancer cells’ colony-forming ability.

### 3.4. Proapoptotic Ability of N. nuda Extract

**Figure 5:**
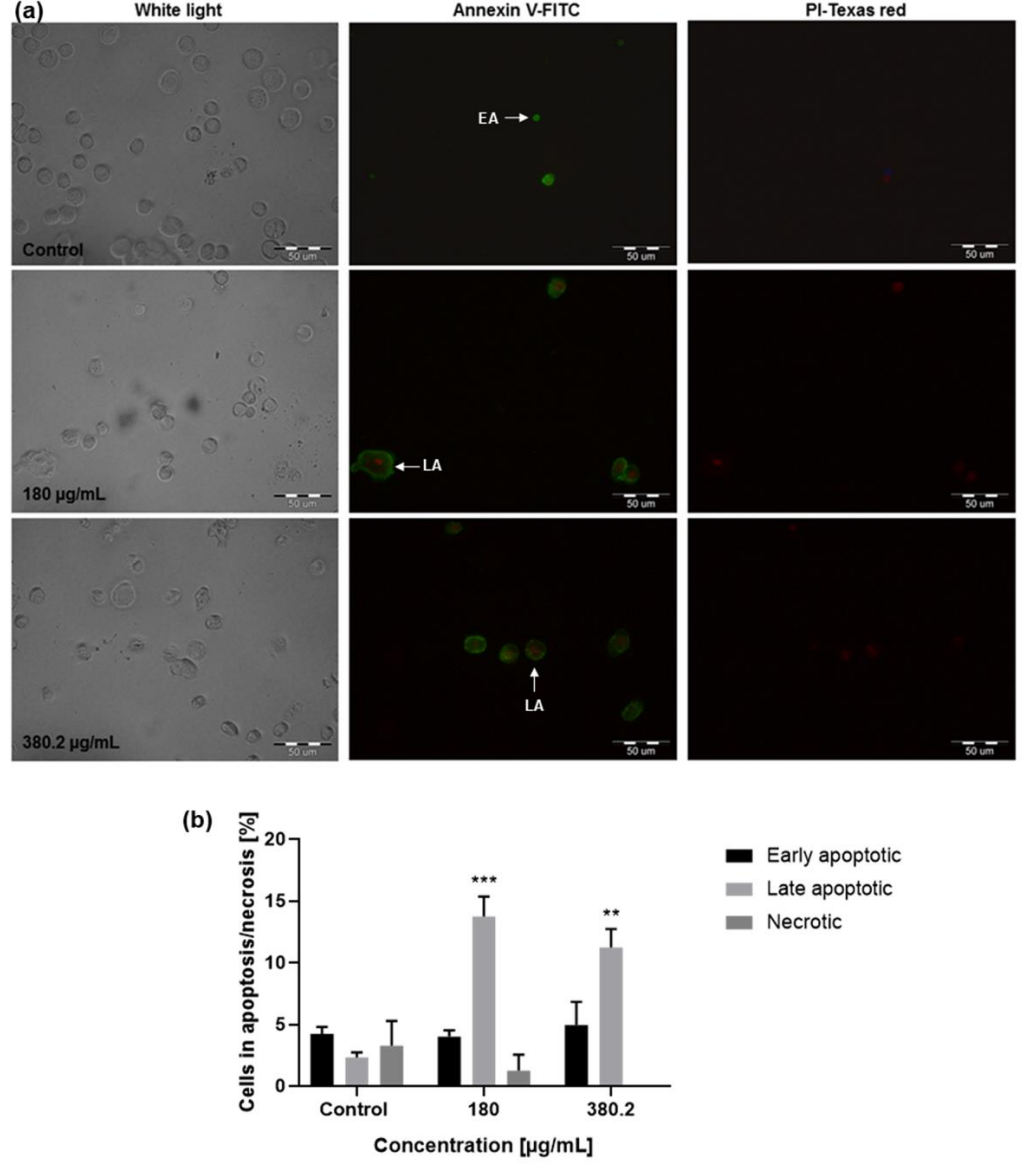
Proapoptotic effect of *N. nuda* aqueous leaf extract. (**a**) Fluorescent microscopy analysis of Colon 26 cells after Annexin V/PI staining for visualization of apoptotic (EA—early apoptotic; LA—late apoptotic) and necrotic cells: the untreated control and extract treated cells (180 and 380.2 μg/mL, 72 h). (**b**) Quantitative evaluation of apoptosis and necrosis. Error bars represent standard error of the mean (SEM). ** *p* < 0.01, *** *p* < 0.001 vs. the control group. Magnification 40×; Scale bar 50 μm.

The fluorescence microscopy assay for the examination of proapoptotic properties of the medicinal plant *N. nuda* in relation to Colon 26 cells was carried out after staining with the dyes Annexin V and propidium iodide (PI), which allowed detecting changes in cell morphology associated with different phases of apoptosis (Figure 5).

Cancer cells were treated for 72 h with the extract and the observation under a fluorescent microscope showed induction of considerable late apoptotic events at applied concentrations of 180 (13.76% of the cells) and 380.2 μg/mL (11.26%) when compared to the untreated control (3.27%). No induction of necrosis was detected in Colon 26 cells at the applied extract concentrations.

### 3.5. Influence of N. nuda Extract on the Expression Level of Genes Associated with Programmed Cell Death and Cell Cycle Control

**Figure 6:**
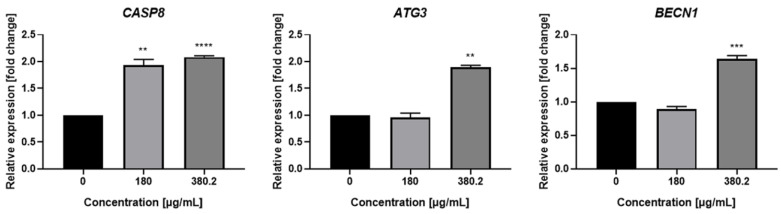
Relative expression levels of *CASP8*, *ATG3* and *BECN1* genes at 72 h after treatment of Colon 26 cells with *N. nuda* aqueous leaf extract at concentrations of 180 and 380.2 μg/mL. Black columns refer to untreated controls. Error bars represent standard error of the mean (SEM); *β-actin* was used as an endogenous control to normalize the expression of the genes. ** *p* < 0.01, *** *p* < 0.001, **** *p* < 0.0001 vs. the control group.

To deepen studies on the targeted molecules and mechanisms of the anticancer action of the aqueous leaf extract of *N. nuda*, the relative expression levels of specific genes with a key role in the process of directing cancer cells to apoptosis and autophagy and in the cell cycle control, were evaluated by quantitative RT-PCR analysis. Transcriptional analysis was performed after treatment of Colon 26 tumor cells with the plant extract for 72 h, and untreated cells incubated for the same time period were used as negative controls, as gene expression levels in the control were taken as 1. The performed transcriptional analysis revealed a statistically significant increase in the expression of *CASP8*, *ATG3* and *BECN1* genes involved in the apoptotic and autophagy pathways (Figure 6).

## 4. Discussion

From the medical perspective, surgery, radiation therapy, hormone therapy, immunotherapy, and chemotherapy are the most common cancer treatment options. However, these approaches are often accompanied by numerous adverse side effects, including hepatotoxicity, nephrotoxicity, low platelet count, alopecia, vomiting, fatigue, and many others. The possibility of introducing new, targeted, relatively safe, and cost-effective cancer treatment alternatives as a preferential option is increasingly desirable. As an aid, medicinal plants provide an extraordinary variety of powerful and health-beneficial mesonutrients.

*Nepeta nuda* is known for its strong allelopathic properties [6], serving to suppress the growth of neighboring plants in competition for resources. Therefore, it is intriguing to investigate the combination of the plant’s compounds displaying antiproliferative effects. Various studies have analyzed the antitumor potential and determined the cytotoxic values of different species of *Nepeta* [20,21,22,23,24,25,26,27,28], but none of them includes *N. nuda* L. ssp. *nuda* or has thoroughly analyzed the mechanisms by which the species exerts its anticancer effects.

In our work, the in vitro cytostatic potential of the aqueous leaf extract of *N. nuda* was evaluated by MTT assay and light microscopy observation after 72 h treatment on a wide panel of tumor cell lines, representing some of the most common and deadly cancer types, and the non-cancerous cell line BJ was used as a control. The time period of 72 h was chosen for the analyses of cell proliferation because of the different doubling time of cell lines used as a model system in the study varying from about 20 to 48 h. *N. nuda* extract decreased cell growth of all studied cancer cell lines and the detected cytostatic effect was most considerable on colon cancer cell line Colon 26 with an IC_50_ value of 380.2 μg/mL. The calculated IC_50_ concentrations of the extract for MDA-MB-231, MCF7, HT29, and HepG2 cells were 481.6 μg/mL, 576.1 μg/mL, 504.2 μg/mL, and 541.4 μg/mL, respectively. A significantly higher IC_50_ concentration value of 1346 μg/mL was established for the non-cancerous cell line BJ, pointing to selectivity in the antiproliferative action of the extract. Although the cytostatic effect of the extract does not meet the criteria for high anticancer activity established by the US National Cancer Institute [29], the substance has high selectivity for cancer cells (SI values > 2 was considered as high selectivity), which is an indication of safety even when applied in higher concentrations, and this is essential for finding novel plant-derived anti-neoplastic agents without side effects.

Based on the extract-specific sensitivity of the Colon 26 cell line and a favorable and higher selectivity index (SI = 3.5) found in the same cell line, it was selected for further investigation. Thereafter, a clonogenic test, fluorescence microscopy, and gene expression analysis were conducted to clarify some aspects and pathways in the anticancer effects of the extract in detail. Our study showed that the analyzed extract had an enviable anti-clonogenic effect against Colon 26 after 7 days of treatment. Furthermore, subsequent treatment for 72 h induced characteristic features of late apoptosis in the Colon 26 cell line, as determined by Annexin V/PI staining. The performed qRT-PCR analysis detected alterations in the relative expression of genes involved in the programmed cell death pathways of apoptosis and autophagy (upregulation of *CASP8*, *ATG3*, and *BECN1*).

The water solvent was shown to assure the highest extract yield from *N. nuda* plant material ~37% dry extract, compared to methanol (~11%), ethanol (~9%), acetone (~4%), and chloroform (~14%) [6]. At 60 °C, the highest total content of phenolics and respective antioxidant activity was measured in aqueous and methanol extracts—nearly 2 to 10 folds more than the use of other solvents. Recent advances suggest the role of natural products including phenolics in photodynamic therapy based on photosensitization (PDT) [30,31]: due to their heterocyclic structure, the phenolic antioxidants act as photosensitizers, and combined with visible light and oxygen, they generate reactive oxygen species (ROSs). High levels of ROSs disrupt cellular homeostasis, damaging lipids, proteins, and DNA, which often triggers apoptosis, necrosis, and autophagy. The balance and extent of these processes vary depending on the type and intensity of oxidative stress, making ROSs a potential target for anticancer therapies that aim to selectively kill cancer cells. In support, in our work, the phenolic antioxidants strongly correlate with the Colon 26 cancer cells’ antiproliferative activity.

The presence of numerous phytoconstituents in the investigated leaf extract of the herb [6] and, in particular, the high content of the polyphenolic compounds rosmarinic acid (RA) and ferulic acid (FA), as well as the iridoid glycoside epideoxyloganic acid, could be a cause of the established antiproliferative and anti-clonogenic effect of *N. nuda*. Available data confirm that RA possesses various pharmacological effects such as antioxidant, anti-inflammatory, antimutagenic, colony-inhibitory, anticancer, etc. [32,33,34,35,36,37,38]. In particular, in colon carcinoma cell lines, CT26 and HCT116, the antimetastatic, antiproliferative, anti-invasive, and anti-migratory activities of RA are mediated by the activation of AMP-activated protein kinase, induction of caspase-3, -8, and -9, as well as reduction of the MMP-2, MMP-9, cyclin D1, CDK4, ICAM-1, and integrin β1 expression [39].

In regard to FA, a considerable body of scientific evidence has also reported the immunoregulatory [40], anti-inflammatory [41], antiproliferative and apoptosis-inducing [42], anti-migratory [43], and anti-invasive [44] activities of the polyphenolic substance. Furthermore, there are data reporting that FA induces autophagy [45] and inhibits multidrug resistance (MDR) in tumor cells [46]. In the case of colon cancer, Chen et al. (2023) [47] found that FA inhibited the growth of CT26WT cells and promoted apoptosis by downregulating *BCL-2* and upregulating the *BAX*, *JNK*, and *ERK* expressions.

Already identified in numerous flowering plants of the genus *Nepeta* [48,49,50,51], as well as in our previous study on *N. nuda* L. *nuda* as the metabolite with the highest amount [6], 1,5,9-epideoxyloganoic acid (1,5,9-eDLA) is known to have various properties, including antibacterial, antioxidant, anti-inflammatory, DNA-protective, and enzyme-inhibitory activities [52,53,54].

This study’s transcriptional analysis of the genes involved in the processes of programmed cell death, apoptosis and autophagy, and cell cycle control found a statistically significant alteration in *CASP8*, *ATG3*, and *BECN1*’s gene expression. Caspase-8 plays a central role in mediating Fas-induced apoptosis [55]. A dose-dependent increase in relative expression levels was found for *CASP8* to 2.09-fold at the highest applied extract concentration of 380.2 μg/mL.

*ATG3* (*Autophagy-related gene 3*) is essential for the autophagy process, acting as an E2 ubiquitin-like conjugating enzyme in the ATG8 conjugation system, contributing to the elongation of the phagophore. *ATG3* also is involved in many physiological and pathological processes in an autophagy-dependent manner, such as tumor progression, maintaining mitochondrial homeostasis, etc. [56,57]. A 1.89-fold increase was observed in the transcriptional levels of *ATG3* after the cancer cells treatment with *N. nuda* extract.

BECN1 (Beclin 1) plays a key role in autophagy initiation, autophagosome formation, and maturation [58]. It is found that in colon cancer the significantly lower expression of *BECN1* compared with the normal colon tissue was positively associated with poor prognosis in patients [59]. In our work, upregulation of *BECN1* was detected with a 1.64-fold increase in the expression levels.

The current study provides information on the very poorly studied antitumor properties of *N. nuda* and represents a comprehensive assessment of some mechanisms of the antitumor potential of the plant.

## 5. Conclusions

In this study, we demonstrated that the aqueous leaf extract of wild-grown medicinal plant *N. nuda* possesses antiproliferative properties against breast, colon, and liver cancer cell lines, and the strongest inhibitory effect is detected against Colon 26 cancer cells. The growth of the non-cancerous cell line BJ was affected to a considerably lower degree. Regarding Colon 26 cells, the extract significantly inhibited the ability of cells to form colonies, exhibited considerable proapoptotic potential involving the participation of the *CASP8* gene, and affected the expression levels of *ATG3* and the *BECN1* gene, possibly involving autophagic cell death among the mechanisms underlying its antitumor action. Future research will be directed to a more detailed study on the molecular targets of the anticancer action of *N. nuda* extract. Biotechnological strategies, including the optimization of plant extraction conditions and the use of in vitro plant cultures, will be tested to improve the antitumor effect. Efforts will be made to identify whether a more specific phytochemical fraction would retain the bioactive potential.

## Figures and Tables

**Table 1:** The IC_50_ values determined by MTT assay and selectivity index.

Cell Line	IC_50_ [μg/mL]	SI
MDA-MB-231	481.6	2.8
MCF7	576.1	2.3
HT29	504.2	2.7
**Colon 26**	**380.2**	**3.5**
HepG2	541.4	2.5
BJ	1346	

## Data Availability

Data presented in this study are available on request from the corresponding author.

## Supplementary Materials

The following supporting information can be downloaded at: https://www.mdpi.com/article/10.3390/life14121539/s1, Table S1: Secondary metabolites identified by Orbitrap-MS^n^ analysis in *N. nuda* leaves [6]; Table S2: Correlation between quantitative parameters of the aqueous extract from [6] and the antitumor properties (IC_50_; Table 1); Figure S1: Quantitative representation of metabolites in leaves of *N. nuda* by using UHPLC/MS2 analysis [6].
