# Supplementary material for: In Vitro Anticancer Effects of Aqueous Leaf Extract from Nepeta nuda L. ssp. nuda
Source: Life (Basel). 2024 Nov 24;14(12):1539. doi: 10.3390/life14121539 (PMC11678516; doi:10.3390/life14121539)
Supplement: Supplementary file 1 [file life-14-01539-s001.zip › life-3278302-supplementary.pdf]

Table S1. Secondary metabolites identified by Orbitrap-MS^n analysis in *N. nuda* leaves [6].

Phenolic acid derivatives	Flavonoids	Iridoid glycosides
Aesculetin	Apigenin	Epideoxyloganic acid 1
Aesculin	Apigenin 7-*O*-(acetyl)hexuronide	Epideoxyloganic acid 2
Benzoyl tartaric acid	Apigenin 7-*O*-(acetyl-caffeoyl)hexuronide	Epideoxyloganic acid 3
Caffeic acid	Apigenin 7-*O*-(caffeoyl)hexuronide	Epideoxyloganic acid hexoside 1
Caffeic acid hexoside 1	Apigenin 7-*O*-(feruloyl)hexuronide 1	Epideoxyloganic acid hexoside 2
Caffeic acid hexoside 2	Apigenin 7-*O*-(feruloyl)hexuronide 2	Epideoxyloganic acid pentoside
Caffeic acid hexuronide	Apigenin 7-*O*-(sinapoyl)hexuronide	Geniposidic acid
Caffeoyl tartaric acid	Apigenin 7-*O*-hexoside	Loganic acid
Clinopodic acid A	Apigenin 7-*O*-hexuronide	Nepetanudoside
Dihydroxybenzoic acid hexoside 1	Apigenin 7-*O*-hexuronide methyl ester	Nepetanudoside B
Dihydroxybenzoic acid hexoside 2	Apigetrin	
Dihydroxybenzoic acid hexoside 3	Astragalin	
Ethyl caffeate	Cirsimaritin	
Ferulic acid	Galangin	
Feruloyl tartaric acid	Isoquercetin	
Gallic acid hexoside 1	Luteolin	
Gallic acid hexoside 2	Luteolin-7-O-diglucuronide	
Gentisic acid	Luleolin 7-*O*-(feruloyl)hexuronide 1	
Methyl 2-hydroxy-3-(3-hydroxy-4-methoxyphenyl) propanoate	Luleolin 7-*O*-(feruloyl)hexuronide 2	
Methyl rosmarinate	Luleolin 7-*O*-hexuronide 1	
Methyl salvianolate C 1	Luleolin 7-*O*-hexuronide 2	
Methyl salvianolate C 2	Luteolin 7-*O*-(caffeoyl)hexuronide	
Nepetoidin B 1	Luteolin 7-*O*-(acetyl)hexuronide 1	
Nepetoidin B 2	Luteolin 7-*O*-(acetyl)hexuronide 2	
p-Hydroxybenzoic acid	Thymusin	
Protocatechuic acid	Xanthomicrol	
Salvianolic acid C		
Rosmarinic acid		
Syringic acid		
Vanillic acid		

Table S2. Correlation between quantitative parameters of the aqueous extract from [6] and the antitumor properties (IC_{50}; Table 1). The asterisk indicates p<0.0.5.

	IC_{50} MDA-MB-231	IC_{50} MCF7	IC_{50} HT29	IC_{50} Colon 26	IC_{50} HepG2
Phenols	0.710	-0.151	0.358	**-0.907***	0.604
Flavonoids	-0.604	0.830*	-0.039	-0.238	-0.534
DPPH	0.837*	-0.447	0.320	-0.682	0.720

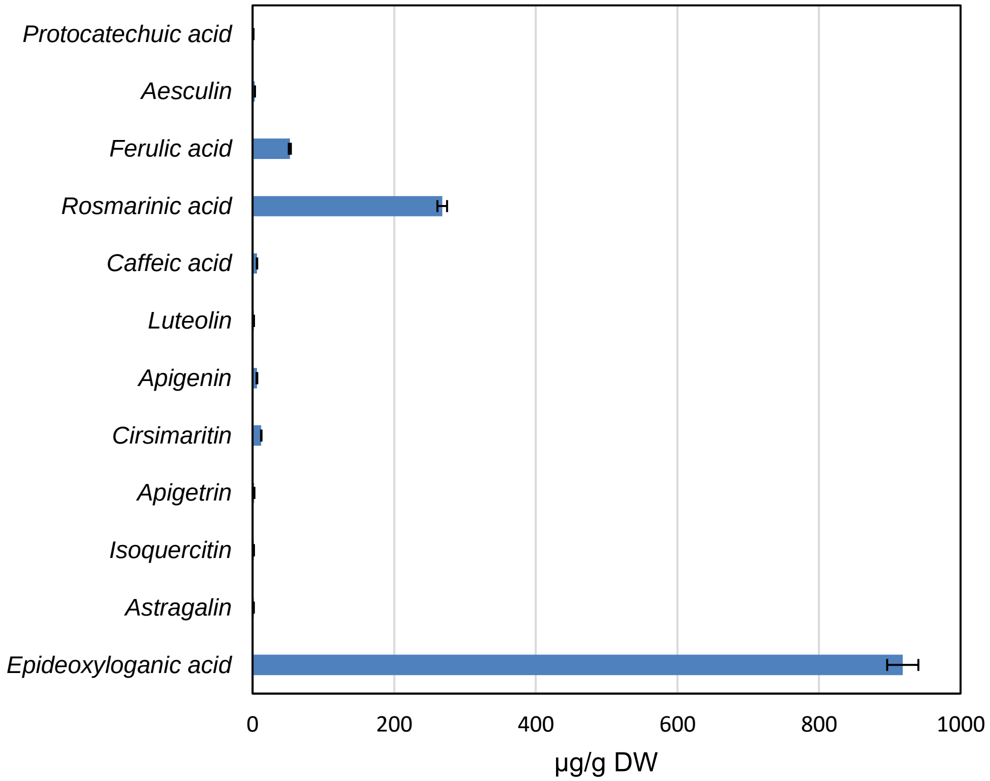

Figure S1. Quantitative representation of metabolites in leaves of *N. nuda* by using UHPLC/MS2 analysis [6].
